# Prognostic Impact of Resection Margin Status in Distal Pancreatectomy for Ductal Adenocarcinoma

**DOI:** 10.1245/s10434-021-10464-6

**Published:** 2021-07-22

**Authors:** Mushegh A. Sahakyan, Caroline S. Verbeke, Tore Tholfsen, Dejan Ignjatovic, Dyre Kleive, Trond Buanes, Kristoffer Lassen, Bård I. Røsok, Knut Jørgen Labori, Bjørn Edwin

**Affiliations:** 1 The Intervention Centre, Rikshospitalet, Oslo University Hospital, Oslo, Norway; 2 Division of Emergencies and Critical Care, Department of Research and Development, Oslo University Hospital, Oslo, Norway; 3 Department of Surgery N1, Yerevan State Medical University, Yerevan, Armenia; 4 Institute of Clinical Medicine, University of Oslo, Oslo, Norway; 5 Department of Pathology, Rikshospitalet, Oslo University Hospital, Oslo, Norway; 6 Department of Hepato-Pancreato-Biliary Surgery, Rikshospitalet, Oslo University Hospital, Oslo, Norway; 7 Department of Digestive Surgery, Akershus University Hospital, Lørenskog, Norway; 8 Institute of Clinical Medicine, University of Tromsø, Tromsø, Norway

## Abstract

**Background:**

Resection margin status is considered one of the few surgeon-controlled parameters affecting prognosis in pancreatic ductal adenocarcinoma (PDAC). While studies mostly focus on resection margins in pancreatoduodenectomy, little is known about their role in distal pancreatectomy (DP). This study aimed to investigate resection margins in DP for PDAC.

**Methods:**

Patients who underwent DP for PDAC between October 2004 and February 2020 were included (*n* = 124). Resection margins and associated parameters were studied in two consecutive time periods during which different pathology examination protocols were used: non-standardized (period 1: 2004–2014) and standardized (period 2: 2015–2020). Microscopic margin involvement (*R*1) was defined as ≤1 mm clearance.

**Results:**

Laparoscopic and open resections were performed in 117 (94.4%) and 7 (5.6%) patients, respectively. The *R*1 rate for the entire cohort was 73.4%, increasing from 60.4% in period 1 to 83.1% in period 2 (*p* = 0.005). A significantly higher *R*1 rate was observed for the posterior margin (35.8 vs. 70.4%, *p* < 0.001) and anterior pancreatic surface (based on a 0 mm clearance; 18.9 vs. 35.4%, *p* = 0.045). Pathology examination period, poorly differentiated PDAC, and vascular invasion were associated with *R*1 in the multivariable model. Extended DP, positive anterior pancreatic surface, lymph node ratio, perineural invasion, and adjuvant chemotherapy, but not *R*1, were significant prognostic factors for overall survival in the entire cohort.

**Conclusions:**

Pathology examination is a key determinant of resection margin status following DP for PDAC. A high *R*1 rate is to be expected when pathology examination is meticulous and standardized. Involvement of the anterior pancreatic surface affects prognosis.

**Supplementary Information:**

The online version contains supplementary material available at 10.1245/s10434-021-10464-6.

Surgery remains the cornerstone of treatment for pancreatic cancer.^1^ Resection margin status is considered one of the few surgeon-controlled parameters affecting prognosis.^2^–^4^ Hence, surgeons strive to improve the quality of surgery to avoid positive resection margins (*R*1). At the same time, the reported *R*1 rate for pancreatectomy lies between 17% and 85%.^5^ On the one hand, the wide variation is attributed to different definitions used for *R*1,^5^,^6^ and, on the other hand, recent studies suggest that the meticulousness of the pathology work-up significantly influences the incidence of *R*1.^7^–^9^ Therefore, the *R*1 rate can also be regarded as a quality marker for pathology.

Multiple studies have addressed the issue of resection margins in pancreatic cancer surgery,^10^–^13^ and while these studies focus on pancreatoduodenectomy, little is known about resection margin status and its clinical relevance in the case of distal pancreatectomy (DP). The latter is a less common procedure for pancreatic cancer compared with pancreatoduodenectomy. Furthermore, resection margins in DP are not the same given the differences in anatomy and resected structures in both specimen types. Thus, examination of the resection margins in DP requires, to some extent, a different approach. Most importantly, while pathology examination protocols for DP specimens have been published,^14^ there is currently no international consensus.

This study examines resection margin status in DP specimens with ductal adenocarcinoma. Pathology examination methods, factors associated with *R*1, and the impact of margin status on patient prognosis are investigated.

## Methods

Patients operated for pancreatic ductal adenocarcinoma (PDAC) in the body or tail of the pancreas at Oslo University Hospital, Rikshospitalet, between October 2004 and February 2020, were included in this study. All patients were evaluated by the multidisciplinary team before being referred to surgery, and patients without preoperative radiological evidence of tumor invasion into adjacent major vessels were referred to laparoscopic DP. Tumor invasion into adjacent organs necessitating an extended DP was not considered a contraindication for a laparoscopic approach. Patients with preoperative radiological suspicion of tumor invasion into major vessels were referred to open surgery.

Neoadjuvant chemotherapy was not administered to patients with primary resectable PDAC. Those with borderline resectable/locally advanced PDAC were treated with preoperative chemotherapy (preferably FOLFIRINOX). Patients who eventually underwent DP following preoperative chemotherapy were included in this study. In highly selected patients in whom a single distant metastasis or limited peritoneal spread was detected intraoperatively, synchronous metastasectomy was performed. Surgical technique, adjuvant therapy, and follow-up strategy have been described elsewhere.^15^–^17^

### Study Design and Exclusion Criteria

Data on patient demographics, clinical characteristics, perioperative outcomes, and pathology findings were retrieved from a prospectively maintained database. Resection margin status and other pathology parameters were studied in two consecutive time periods during which different approaches to pathology examination were used: non-standardized (period 1: 2004–2014) and standardized (period 2: 2015–2020). Factors associated with an *R*1 resection were investigated. Patients with incomplete data (*n* = 14) and those participating in ongoing randomized controlled trials (*n* = 10) were excluded from the analysis, as were patients with tumors other than PDAC or its subtypes,^18^ as well as those with adenocarcinoma associated with intraductal papillary mucinous neoplasia.

The impact of resection margin status on long-term oncologic outcomes (recurrence and survival) was evaluated in patients with non-metastatic PDAC. Those with limited, intraoperatively detected metastatic disease were excluded from these analyses. Survival data were obtained from the Norwegian National Population Registry, and local hospitals were contacted, when necessary. The study was approved by the Hospital Review Board according to the guidelines provided by the Regional Ethics Committee. For patients not included in the Thematic Pancreatic Tumour Project (REK ref. 2015/738), the Regional Ethics Committee waived written consent (REK Sør-Øst B 2018/1060) for those patients who had died at date of last follow-up.

This study was performed according to the Strengthening the Reporting of Observational Studies in Epidemiology (STROBE) guidelines.^19^ Comprehensive information on the distribution of patients’ race or ethnicity was not available due to the retrospective nature of this study.

### Specimen Grossing

During period 1, grossing of DP specimens was performed by trained technical laboratory staff. Following fixation and color-coded inking of the anterior and posterior specimen surfaces, the specimen was sliced either along the longitudinal axis of the pancreatic body/tail, in the sagittal plane, or a combination of both, as described previously.^20^ Samples were taken from the tumor-bearing pancreas and from palpated peripancreatic or hilar lymph nodes. In the case of extended DP specimens, dissection and tissue sampling was left to the discretion of the laboratory staff and supervising consultant pathologist.

During period 2, specimen grossing was performed by a specialist consultant pathologist (CSV) following a procedure that has been described previously.^21^,^22^ The anterior and posterior surfaces of the pancreas, as well as the transection margins of the splenic artery and vein, were inked in different colors. The specimen was thinly sliced (3 mm slice thickness) in the sagittal plane. Numerous tissue blocks were taken from the grossly visible tumor onto the specimen surfaces. All visible lymph nodes were completely embedded, and peripancreatic or hilar adipose tissue that was not clearly devoid of lymph nodes was extensively sampled. In the case of neoadjuvant treatment, all tissues and structures that were not entirely normal were embedded. The same dissection and sampling procedures were followed for extended resection specimens, with extra tissue samples being taken from the tumor onto the additionally resected structures and their relevant surfaces, in particular circumferential resection margins (e.g., the surface of the soft tissue plane between the tumor and left adrenal).

### Definitions

Standard and extended DP, as well as concomitant (non-contiguous organ) resections were defined according to the consensus criteria suggested by the International Study Group for Pancreatic Surgery (ISGPS).^23^ The Accordion Severity Grading System was applied to classify postoperative complications;^24^ grade III or higher complications were considered severe. Postoperative pancreatic fistula (POPF) and hemorrhage were defined and graded according to the ISGPS.^25^,^26^

Tumor size was defined as the largest diameter reported on pathology, and *R*1 was defined as a 1 mm or less clearance to a margin and 0 mm clearance to the anterior pancreatic surface. Lymph node ratio was calculated by dividing the number of positive lymph nodes by the total number of examined lymph nodes. TNM stage was determined based on the 8th edition of the American Joint Committee on Cancer/Union for International Cancer Control (AJCC/UICC) staging system for pancreatic cancer.^27^,^28^

Recurrence was defined as radiological evidence of intra-abdominal soft tissue around the surgical site and/or distant metastases, and was classified as local recurrence, distant (hematogenous) metastasis, or peritoneal carcinomatosis. Patients without recurrence were censored at the last follow-up. Overall survival was estimated from the date of surgery until the date of death or the date of censoring (1 December 2020).

### Statistics

Continuous data were expressed as median (range) or mean (± standard deviation), while frequencies (percentages) were applied for categorical data. The two-sample Student’s *t*-test and Mann–Whitney *U* test were used for normally and non-normally distributed continuous variables, respectively, and the Chi-square and Fisher’s exact tests were applied for the frequencies. A two-tailed *p*-value < 0.05 was considered statistically significant. A multivariable binary logistic regression model was used to examine the association between *R*1 and clinicopathological parameters that were significant in the univariable analysis.

The Kaplan–Meier method was used to estimate survival and to plot survival curves, with survival being described as median (95% confidence interval). Univariable and multivariable Cox regression analyses were applied to identify prognostic factors for survival. Parameters significant at *p* < 0.1 in the univariable analysis were added to the multivariable model with backward selection. A two-tailed *p*-value <0.05 was considered statistically significant.

## Results

### Perioperative Results

Overall, 124 patients underwent DP for PDAC within the study period. Preoperative chemotherapy was administered to 9 (7.3%) patients. Patient characteristics and perioperative data are presented in electronic supplementary Table 1. Standard resections were performed in 68.5% of cases, and laparoscopic DP was initiated in 117 (94.4%) patients, 9 (7.7%) of whom underwent conversion. Fifty-two (41.9%) patients had complications, including 30 (24.2%) patients with severe complications. Ninety-day mortality was 0.8% (1/124) and median length of stay was 5 days (3–49).

### Pathology Findings and Predictors for *R*1

Most patients (54%) had pT3 cancer without a significant difference between both periods. The proportion of node-negative (pN0) specimens decreased from 43.3% in period 1 to 19.7% in period 2 (Table 1), while the incidence of pN2 significantly increased (13.2 vs. 39.4%, *p* < 0.001). The lymph node yield also significantly increased, from 8 in period 1 to 20 in period 2 (*p* < 0.001). During period 2, a significantly larger number of tissue blocks from the tumor and adjacent structures and margins were examined (14 vs. 9, *p* < 0.001).

**Table 1:** R status and other pathology-based tumor features in distal pancreatectomy specimens

Parameter	Total [*n* = 124]	Period 1 [*n* = 53]	Period 2 [*n* = 71]	*p* value
Tumor size, mm [mean (SD)]	43.6 (18.4)	40.9 (17.6)	45.5 (18.8)	0.17
pT stage				0.79
pT1	12 (9.7)	6 (11.3)	6 (8.5)	
pT2	45 (36.3)	20 (37.7)	25 (35.2)	
pT3	67 (54)	27 (50.9)	40 (56.3)	
pN stage				< 0.001
pN0	37 (29.8)	23 (43.4)	14 (19.7)	
pN1	52 (41.9)	23 (43.4)	29 (40.8)	
pN2	35 (28.2)	7 (13.2)	28 (39.4)	
M1 stage	11 (8.9)	5 (9.4)	6 (8.5)	1.0
Lymph node yield [median (range)]	16 (1–48)	8 (1–48)	20 (6–41)	< 0.001
Poor differentiation^a^	38 (33)	11 (22)	27 (41.5)	0.027
Vascular invasion	88 (71)	35 (66)	53 (74.6)	0.29
Lymphatic invasion	84 (67.7)	29 (54.7)	55 (77.5)	0.007
Perineural invasion	110 (88.7)	47 (88.7)	63 (88.7)	1.0
Tumor blocks examined [median (range)]	12 (4–27)	9 (4–22)	14 (6–27)	< 0.001
*R* status				0.005
*R*0	33 (26.6)	21 (39.6)	12 (16.9)	
*R*1	91 (73.4)	32 (60.4)	59 (83.1)	
Positive margins and surfaces				
Transection pancreas	16 (12.9)	8 (15.1)	8 (11.3)	0.53
Posterior	69 (55.6)	19 (35.8)	50 (70.4)	< 0.001
Anterior surface	35 (28.2)	10 (18.9)	25 (35.2)	0.045
Transection splenic artery/vein	7 (5.6)	–^b^	7 (9.9)	–
>1 positive margin/surface	34 (27.4)	6 (11.3)	28 (39.4)	0.001

Data are expressed as *n* (%) unless otherwise specified

*SD* standard deviation

^a^Not applicable to tumors treated with preoperative chemotherapy

^b^Not studied in period 1

The *R*1 rate for the entire cohort was 73.4%, increasing from 60.4% in period 1 to 83.1% in period 2 (*p* = 0.005). Furthermore, the *R*1 rate at the posterior margin increased from 35.8% to 70.4% (*p* < 0.001), and, for the anterior pancreatic surface, the *R*1 rate increased from 18.9% to 35.4% (*p* = 0.045). In addition, involvement of more than one margin or surface also increased significantly (11.3 vs. 39.4%, *p* = 0.001). The pathology examination period, grade of differentiation, tumor size, pT and pN stages, and lymphatic and vascular invasion correlated with *R*1 in the univariable model (Table 2). In multivariable analysis, pathology examination period, poor differentiation, and presence of vascular invasion were independent predictors for *R*1.

**Table 2:** Clinical and pathology parameters associated with *R*1 status following distal pancreatectomy for adenocarcinoma: univariable and multivariable analysis

Parameter	Univariable analysis			Multivariable analysis	
	*R*0 [*n* = 33]	*R*1 [*n* = 91]	*p*-Value	OR (95% CI)	*p*-Value
Age, years [mean (SD)]	67.7 (9.8)	66.7 (9.9)	0.6		
Body mass index [mean (SD)]	23.8 (4.2)	25.5 (4.6)	0.07		
Male sex	19 (57.6)	53 (58.2)	0.95		
Preoperative chemotherapy	2 (6.1)	7 (7.7)	1.0		
Surgeon (senior consultant)	27 (81.8)	71 (78)	0.65		
Standard resection	27 (81.8)	58 (63.7)	0.06		
Laparoscopic procedure	33 (100)	84 (92.3)	0.19		
Conversion^a^	1 (3)	8 (9.5)	0.44		
Standardized pathology work-up	12 (36.4)	59 (64.8)	0.005	2.9 (1.14–7.2)	0.025
Poor differentiation^b^	4 (12.9)	34 (40.5)	0.006	3.4 (1.03–11.4)	0.045
Tumor size, mm [mean (SD)]	36.8 (18.2)	46 (18)	0.014	–	NS
pT stage			0.019		
pT1	7 (21.2)	5 (5.5)		Reference	–
pT2	13 (39.4)	32 (35.2)		–	NS
pT3	13 (39.4)	54 (59.3)		–	NS
pN stage			<0.01		
*N*0	18 (54.5)	19 (20.9)		Reference	–
*N*1	12 (36.4)	40 (44)		–	NS
*N*2	3 (9.1)	32 (35.2)		–	NS
Splenic vein invasion^c^	4 (28.6)	34 (56.7)	0.06		
Splenic artery invasion^c^	0 (0)	5 (8.3)	0.58		
Lymphatic invasion	17 (51.5)	67 (73.6)	0.02	–	NS
Vascular invasion	15 (45.5)	73 (80.2)	< 0.01	3.65 (1.44–9.3)	0.006
Perineural invasion	28 (84.8)	82 (90.1)	0.52		

Data are expressed as *n* (%) unless otherwise specified

*OR* odds ratio, *CI* confidence interval, *NS* non-significant, *SD* standard deviation

^a^Calculated for laparoscopic cases

^b^Not applicable in patients treated with preoperative chemotherapy

^c^Invasion into the splenic vein/artery was not reported in period 1; c-statistic = 0.84

### Long-Term Oncologic Outcomes

After excluding patients with distant metastasis (*n* = 11), 113 patients with PDAC were analyzed for long-term oncologic outcomes, of whom 34 (30.1%) underwent extended DP and 79 (69.9%) had standard DP (23 *R*0 and 56 *R*1). R status and positive resection margins/surfaces in extended DP specimens are presented in electronic supplementary Table 2.

Median follow-up was 19 months (3–108). Patients were comparable in terms of the administration of preoperative and adjuvant chemotherapy (Table 3). Recurrence was observed in 86 (76.1%) patients. Extended DP resulted in a significantly higher incidence of recurrence compared with *R*0 standard and *R*1 standard DP (91.2 vs. 69.6 vs. 69.6%, *p* = 0.048). No statistically significant association was found between the recurrence site and R status/extent of surgery. The site of resection margin/surface involvement did not correlate with either the development of recurrence or the site of recurrence (electronic supplementary Table 3).

**Table 3:** Chemotherapy and disease recurrence in patients with non-metastatic ductal adenocarcinoma undergoing standard (*R*0/*R*1) and extended distal pancreatectomy

Parameter	Standard *R*0 [*n* = 23]	Standard *R*1 [*n* = 56]	Extended [*n* = 34]	*p*-Value
Preoperative chemotherapy	0 (0)	5 (8.9)	1 (2.9)	0.33
Adjuvant chemotherapy	18 (78.3)	35 (62.5)	22 (64.7)	0.39
Recurrence	16 (69.6)	39 (69.6)	31 (91.2)	0.048
Local	6 (26.1)	18 (32.1)	11 (32.4)	0.85
Distant metastases	11 (47.8)	24 (42.9)	19 (55.9)	0.49
Peritoneal carcinomatosis	2 (8.7)	7 (12.5)	10 (29.4)	0.08

Data are expressed as n (%)

**Fig. 1:**
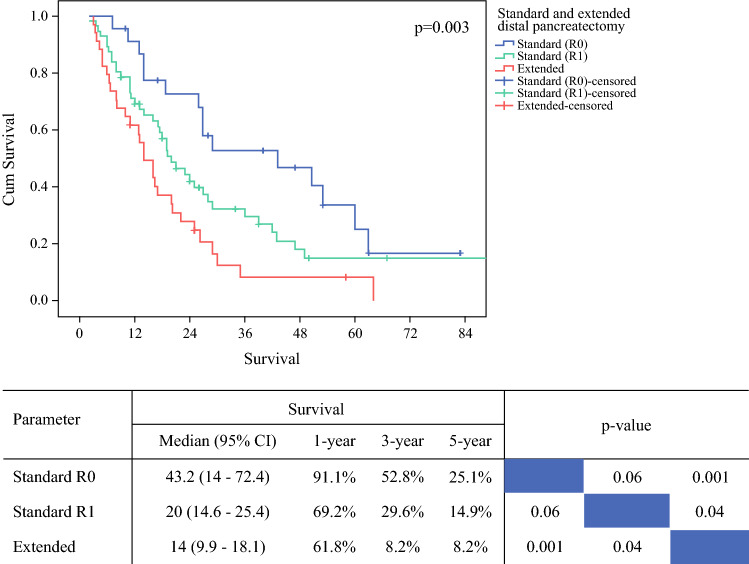
Survival Following standard (*R*0/*R*1) and extended distal pancreatectomy in patients with non-metastatic ductal adenocarcinoma. *Cum* cumulative, *CI* confidence interval

Overall median survival was 20 months (15.2–24.8). Extended DP was associated with a significantly shorter median survival compared with standard DP (*R*1 and *R*0), i.e. 14 vs. 20 vs. 43.2 months (*p* = 0.003) (Fig. 1). Univariable analysis revealed that extended resection, perioperative red blood cell transfusion, pN stage, lymph node ratio, resection margin status, anterior pancreatic surface, number of resection margins/surfaces involved, vascular and perineural invasion, and adjuvant chemotherapy were associated with survival (Table 4). In the multivariable model, extended resection, anterior pancreatic surface, lymph node ratio, perineural invasion, and adjuvant chemotherapy, but not overall resection margin status, were predictors for survival. Prognostic factors in different study periods are presented in electronic supplementary Table 4. *R*1 was statistically significant in the univariate analysis of period 2, but not in the multivariable analysis.

**Table 4:** Univariable and multivariable analysis of prognostic factors for overall survival following distal pancreatectomy for non-metastatic ductal adenocarcinoma

Variables	Univariable		Multivariable	
	HR (95% CI)	*p* value	HR (95% CI)	*p* value
Age, years	1.01 (0.98–1.03)	0.82		
Body mass index, kg/m^2^	0.98 (0.94–1.03)	0.49		
Male sex (vs. female)	0.92 (0.59–1.41)	0.69		
Period 2013–2020 (vs. 2004–2012)	0.86 (0.54–1.39)	0.54		
Red blood cell transfusion	1.67 (0.97–2.90)	0.07	–	–
Extended resection (vs. standard)	1.95 (1.24–3.06)	0.004	2.03 (1.27–3.24)	0.003
Severe complications	1.18 (0.72–1.96)	0.51		
Poor differentiation (vs. well/moderate)	1.22 (0.78–1.91)	0.38		
Tumor stage (vs. pT1)				
pT2	1.46 (0.64–3.33)	0.37		
pT3	1.84 (0.83–4.08)	0.13		
Nodal stage (vs. pN0)				
pN1	1.7 (0.99–2.94)	0.06	–	–
pN2	2.46 (1.38–4.41)	0.002	–	–
Lymph node yield	1.01 (0.99–1.03)	0.46		
Lymph node ratio (increase by 0.01)	1.03 (1.02–1.04)	0.001	1.03 (1.01–1.04)	0.001
*R*1 (vs. *R*0)	1.72 (1.04–2.84)	0.03	–	–
Positive transection margin	1.34 (0.71–2.55)	0.37		
Positive posterior margin	1.1 (0.71–1.69)	0.66		
Positive anterior surface	2.49 (1.57–3.94)	0.001	2.03 (1.26–3.26)	0.004
>1 positive margin/surface	1.8 (1.1–2.96)	0.02	–	–
Splenic vein invasion	1.49 (0.81–2.72)	0.2		
Lymphatic invasion	1.44 (0.91–2.28)	0.12		
Vascular invasion	1.98 (1.2–3.26)	0.007	–	–
Perineural invasion	2.86 (1.24–6.59)	0.014	3.9 (1.6–9.4)	0.003
Adjuvant chemotherapy	0.54 (0.35–0.83)	0.006	0.41 (0.25–0.66)	0.001

c-statistic = 0.74

*HR* hazard ratio, *CI* confidence interval

## Discussion

In this series from a high-volume tertiary referral center of pancreatic surgery, the rate of microscopic margin involvement following DP for PDAC was 73.4% during the entire study period (2004–2020). However, following the introduction of a detailed standardized pathology examination procedure for specimen grossing in 2015, the *R*1 rate increased significantly, from 60.4% to 83.1%. Concomitantly, the incidence of microscopic involvement of the posterior resection margin and the anterior pancreatic surface increased significantly. Given the differences observed after the implementation of the standardized pathology work-up, we cautiously assume that the exact *R*1 rate during the entire study period was higher than 73.4%. The accuracy of the standardized specimen examination also had an impact on the lymph node yield and nodal status, as the number of detected lymph nodes and the rate of positive lymph nodes were significantly higher in period 2, which is in line with a previous report from our group.^20^

An important question to address is whether the *R*1 rate solely relates to the extent of surgery or whether it also reflects the quality of the pathology examination. In our center, neither the surgical technique nor the indications for DP in PDAC changed during the study period. Furthermore, the workload was similar and the same consultant surgeons were involved in both study periods. Therefore, the significant increase in the *R*1 rate observed in this study is likely to be attributed to the implementation of a meticulous, standardized pathology work-up rather than to any aspect of surgery. This is also confirmed in the multivariable analysis demonstrating that the pathology examination period is a significant factor associated with *R*1.

Previous series on DP for PDAC have reported *R*1 rates that are considerably lower and vary significantly, from 0% to 59%.^29^–^33^ At the same time, data provided in these studies are quite heterogenous. A probable explanation for the wide variation is the lack of consensus as to which specimen surfaces are to be included in the assessment of the margin status. Furthermore, different definitions for *R*1 have been used (0 mm or ≤1 mm clearance). Recent studies based on the definitions of *R*1 according to the Royal College of Pathologists (≤1 mm clearance; 0 mm clearance at the anterior surface) report on microscopic margin involvement in 40–45% of DP specimens, which is markedly lower than in our series.^32^,^34^,^35^ However, the data are difficult to compare as the pathology examination protocols that were followed are hardly mentioned in these studies. Furthermore, since these were multicenter studies, the pathology examination methods may not have been uniform between the centers. In contrast, we have used a standardized pathology work-up (in period 2).

A key feature of the standardized grossing protocol used in period 2 is the emphasis on extensive tissue sampling. Indeed, this aspect is hardly ever mentioned in pathology protocols, including in those used for multicenter studies. Likewise, (inter-)national pathology guidelines do not provide any recommendations regarding the extent of sampling, and yet this is a decisive factor when it comes to the detection of *R*1, i.e. *microscopic* margin/surface involvement. As this is, by definition, a microscopic finding and the invasive front of pancreatic cancer is notoriously difficult to identify on naked-eye inspection, more extensive tissue sampling from the tumor onto the adjacent specimen surface increases the likelihood of the detection of microscopic margin involvement. A positive correlation between the number of such tissue samples and the *R*1 rate has been previously described for pancreatoduodenectomy specimens.^8^ In this study, the introduction of a more elaborate specimen examination procedure resulted in a significant increase in the median number of tissue blocks that were examined, i.e. 14 versus 9 in period 1 (*p* < 0.001). Given that the significant increase in the *R*1 rate was observed concomitantly with the use of the new grossing protocol in 2015, we assume that the increased extent of sampling resulted in a higher detection rate of microscopic margin involvement in DP specimens.

One of the main objectives of this study was to examine the impact of *R*1 resection on survival. Standard DP was associated with a significantly longer survival compared with extended DP regardless of the margin status of the former. *R*1 was a significant prognostic factor for survival in the univariable analysis and in the subgroup analysis of patients from study period 2. However, *R*1 was not an independent predictor for survival, unlike factors such as the extent of surgery, anterior pancreatic surface status, lymph node ratio, perineural invasion, and adjuvant chemotherapy. In quantitative terms, the number of tumor cells in transit, either along the lymphatic or peripheral nerve system, is likely to be considerably higher than in one or two discrete foci with *microscopic* residual disease (*R*1). Hence, lymph node involvement and perineural invasion are probably stronger determinants of outcome, whereas *R*1 may be only relevant for long-term outcome in node-negative patients without perineural invasion. However, because this patient group is very small, the establishment of a cohort sufficiently large to dissect out the prognostic impact of *R*1 in these cases seems unrealistic.

To the best of our knowledge, this is the first study to report on the impact of the involvement of the anterior pancreatic surface on survival in patients undergoing DP for PDAC, however its prognostic role in pancreatic head cancer has been previously mentioned.^36^,^37^ Nonetheless, it did not correlate with either the development of recurrence or the site of recurrence, although a trend towards an increased risk of recurrence was seen in cases with involvement of the anterior surface. Our findings also demonstrate that involvement of the anterior pancreatic surface is associated with a higher pN stage, the presence of vascular invasion, involvement of more than one margin/surface, and poor tumor differentiation (electronic supplementary Table 5). Therefore, breaching the peritoneal lining of the pancreas by tumor cells may indicate a more aggressive tumor biology and/or a more advanced stage. Hence, specific reporting of involvement of the anterior surface rather than including it in the overall *R*1 status without further specification may be relevant to the prediction of patient outcome. It should also be borne in mind that the anterior pancreatic surface is unaffected by the extent of surgery and that its involvement is rather determined by the location of the tumor. These findings indicate that neoadjuvant therapy may be considered when involvement of the anterior pancreatic surface and/or tumor ingrowth into adjacent organs is suggested on preoperative imaging. At the same time, it is worth mentioning that in seven of nine patients in this series who had received preoperative chemotherapy, DP resulted in an *R*1 resection. This finding is in line with observations by others.^38^

The prognostic roles of lymph node ratio, perineural invasion, and adjuvant chemotherapy in pancreatic body/tail cancer have been reported previously.^34^,^39^,^40^ The dismal prognosis for patients undergoing extended DP for PDAC has been highlighted in our previous reports and in other studies.^34^,^40^–^43^ In this study, resection margins were positive in 82% of patients undergoing extended resection. Considering that all extended resection specimens that were examined according to the standardized protocol in period 2 showed *R*1, we believe that *R*0 can hardly be expected in this group. Nearly two-thirds of these patients had a positive posterior margin, while in over half, more than one margin/surface was involved (electronic supplementary Table 2). Furthermore, the rates of involvement of the anterior pancreatic surface and of more than one positive margin/surface were significantly higher compared with standard DP.

This study has several limitations. First and foremost, despite the prospectively collected database, this was an observational cohort study with its inherent weaknesses due to retrospectively defined parameters. Second, the findings of this study indicate that *R*1 may have been underreported in period 1, which, in turn, may have influenced our results regarding long-term oncologic outcomes. Third, only very few selected cases underwent open DP, thus there was no possibility to assess the potential influence of surgical approach on resection margin status. Lastly, due to the small number of patients who were treated with preoperative chemotherapy, the impact of the latter on resection margin status and prognosis could not be thoroughly evaluated.

## Conclusion

A meticulous, standardized procedure for specimen grossing is key for the adequate assessment of margin status in DP specimens with PDAC. The systematic use of such a procedure shows that microscopic margin involvement occurs in the vast majority of cases. While the overall R status does not affect prognosis, involvement of the anterior pancreatic surface, extended DP, lymph node ratio, perineural invasion, and adjuvant chemotherapy are associated with survival.

## Supplementary Information

Below is the link to the electronic supplementary material.

Supplementary file1 (DOCX 30 kb)
