# Supplementary material for: Prognostic Impact of Resection Margin Status in Distal Pancreatectomy for Ductal Adenocarcinoma
Source: Ann Surg Oncol. 2021 Jul 22;29(1):366–75. doi: 10.1245/s10434-021-10464-6 (PMC8677636; doi:10.1245/s10434-021-10464-6)
Supplement: Supplementary file 1 — Supplementary file1 (DOCX 30 kb) [file 10434_2021_10464_MOESM1_ESM.docx]

**Supplementary table 1. Baseline characteristics and perioperative data in patients with ductal adenocarcinoma undergoing distal pancreatectomy at Oslo University Hospital, Rikshospitalet.**

| **Variables** | **n=124** |
| --- | --- |
| Age, median (range) | 68 (46-87) |
| Body mass index, median (range) | 24.9 (14-35.7) |
| Female gender, n (%) | 52 (41.9%) |
| Total number of comorbidities, median (range) | 1 (0-6) |
| ASA score ≥ III, n (%) | 52 (43%) |
| Preoperative chemotherapy, n (%) | 9 (7.3%) |
| Tumor size (mm), median (range) | 42.5 (9-88) |
| Surgical approach, n (%) |  |
| Laparoscopic | 117 (94.4%) |
| Open | 7 (5.6%) |
| Type of resection, n (%) |  |
| Standard | 85 (68.5%) |
| Extended | 34 (27.4%) |
| Concomitant | 5 (4.1%) |
| Operative time, min, median (range) | 180 (56-503) |
| Estimated blood loss, ml, median (range) | 150 (50-3000) |
| Red blood cell transfusion, n (%) | 17 (13.9%) |
| Conversion, n (%) * | 9 (7.7%) |
| Postoperative complications, n (%) | 52 (41.9%) |
| Severe complications, n (%) | 30 (24.2%) |
| Postoperative pancreatic fistula, n (%) | 28 (22.6%) |
| Grade B/C postoperative hemorrhage, n (%) | 10 (8.1%) |
| 90-day mortality, n (%) | 1 (0.8%) |
| Postoperative length of stay, days, median (range) | 5 (3 - 45) |

* calculated for laparoscopic cases.

**Supplementary table 2. R-status and site of R1 in extended distal pancreatectomy specimens with ductal adenocarcinoma (period 1 – 16; period 2 - 18).**

| **R-status** | **Margin/surface type** | **(n=34)** |
| --- | --- | --- |
| R1, n (%) |  | 28 (82.4%) |
|  | Transection margin, n (%) | 8 (23.5%) |
|  | Posterior margin, n (%) | 24 (70.6%) |
|  | Anterior pancreatic surface, n (%) | 15 (44.1%) ^┼^ |
|  | Transection splenic artery/vein, n (%) * | 3 (8.8%) |
|  | > 1 positive margin/surface, n (%) | 18 (52.9%) ^╪^ |

* evaluated only in period 2; ^┼^ significant difference in positive anterior surface rate compared with standard resection (44.1 vs 20.3%, p=0.009); ^╪^ significant difference in >1 positive margin/surface rate compared with standard resection (52.9 vs 11.4%, p=0.001).

**Supplementary table 3. Association between the site of resection margin/surface involvement and site of recurrence.**

| **Variables** | **Positive margin/surface** | | | |
| --- | --- | --- | --- | --- |
|  | **Transection**  **(n=15)** | **Posterior**  **(n=66)** | **Anterior**  **(n=31)** | **> 1 positive margin/**  **surface**  **(n=27)** |
| Recurrence, n (%) | 11 (73.3%) | 51 (77.3%) | 27 (87.1%) | 24 (88.9%) |
| Local, n (%) | 8 (53.3%) | 21 (31.8%) | 10 (32.3%) | 10 (37%) |
| Distant metastases, n (%) | 5 (33.3%) | 30 (45.5%) | 17 (54.8%) | 13 (48.1%) |
| Peritoneal carcinomatosis, n (%) | 2 (13.3%) | 13 (19.7%) | 6 (19.4%) | 5 (18.5%) |

* Patients with distant metastases detected at surgery are excluded from this analysis.

**Supplementary table 4a. Uni-/multivariable analysis of prognostic factors for overall survival in period 1.**

| **Variables** | **Univariable** | | | **Multivariable** | |
| --- | --- | --- | --- | --- | --- |
|  | **HR (95% CI)** | **p-value** | **HR (95% CI)** | | **p-value** |
| Age, years | 1.004 (0.98 – 1.03) | 0.79 |  | |  |
| Body mass index, kg/m^2^ | 0.99 (0.93 – 1.05) | 0.68 |  | |  |
| Male gender (vs female) | 1.07 (0.59 – 1.95) | 0.83 |  | |  |
| Red blood cell transfusion | 2.38 (1.15 – 4.93) | 0.019 | _____________ | | - |
| Extended resection (vs standard) | 2.21 (1.17 – 4.18) | 0.014 | _____________ | | - |
| Postoperative complications | 1.89 (1.01 – 3.53) | 0.046 | _____________ | | - |
| Severe complications | 1.41 (0.69 – 2.86) | 0.34 |  | |  |
| Poor differentiation (vs well/moderate) | 0.76 (0.36 – 1.59) | 0.47 |  | |  |
| Tumor stage (vs pT1) |  |  |  | |  |
| pT2 | 1.24 (0.45 – 3.39) | 0.68 |  | |  |
| pT3 | 2.54 (0.95 – 6.76) | 0.06 | _____________ | | - |
| Nodal stage (vs pN0) |  |  |  | |  |
| pN1 | 2.93 (1.48 – 5.77) | 0.002 | _____________ | | - |
| pN2 | 6.16 (2.22 – 17.12) | < 0.001 | _____________ | | - |
| Harvested lymph nodes | 1.02 (0.99 – 1.05) | 0.12 |  | |  |
| Lymph node ratio (increase by 0.01) | 1.06 (1.03 – 1.08) | < 0.001 | 1.06 (1.03 – 1.08) | | < 0.001 |
| R1 (vs R0) | 1.43 (0.78 – 2.62) | 0.24 |  | |  |
| Positive transection margin | 1.87 (0.81 – 4.32) | 0.14 |  | |  |
| Positive posterior margin | 1.17 (0.63 – 2.16) | 0.63 |  | |  |
| Positive anterior surface | 1.64 (0.76 – 3.53) | 0.21 |  | |  |
| > 1 positive margins/surfaces | 2.4 (0.99 – 5.79) | 0.051 | _____________ | | - |
| Splenic vein invasion | - | - |  | |  |
| Vascular invasion | 2.5 (1.31 – 4.78) | 0.006 | 2.39 (1.21 – 4.73) | | 0.012 |
| Perineural invasion | 1.71 (0.67 – 4.37) | 0.26 |  | |  |
| Adjuvant chemotherapy | 0.58 (0.31 – 1.08) | 0.09 | 0.43 (0.22 – 0.84) | | 0.013 |

c-statistic = 0.78

**Supplementary table 4b. Uni-/multivariable analysis of prognostic factors for overall survival in period 2.**

| **Variables** | **Univariable** | | | **Multivariable** | |
| --- | --- | --- | --- | --- | --- |
|  | **HR (95% CI)** | **p-value** | **HR (95% CI)** | | **p-value** |
| Age, years | 1.003 (0.98 – 1.03) | 0.82 |  | |  |
| Body mass index, kg/m^2^ | 0.98 (0.94 – 1.03) | 0.49 |  | |  |
| Male gender (vs female) | 0.92 (0.59 – 1.41) | 0.69 |  | |  |
| Red blood cell transfusion | 1.67 (0.97 – 2.90) | 0.07 | _____________ | | - |
| Extended resection (vs standard) | 1.95 (1.24 – 3.06) | 0.004 | _____________ | | - |
| Postoperative complications | 1.09 (0.69 – 1.69) | 0.72 |  | |  |
| Severe complications | 1.18 (0.72 – 1.96) | 0.51 |  | |  |
| Poor differentiation (vs well/moderate) | 1.22 (0.78 – 1.91) | 0.38 |  | |  |
| Tumor stage (vs pT1) |  |  |  | |  |
| pT2 | 1.46 (0.64 – 3.33) | 0.37 |  | |  |
| pT3 | 1.84 (0.83 – 4.08) | 0.13 |  | |  |
| Nodal stage (vs pN0) |  |  |  | |  |
| pN1 | 1.7 (0.99 – 2.94) | 0.06 | _____________ | | - |
| pN2 | 2.46 (1.38 – 4.41) | 0.002 | _____________ | | - |
| Harvested lymph nodes | 1.01 (0.99 – 1.03) | 0.46 |  | |  |
| Lymph node ratio (increase by 0.01) | 1.03 (1.02 – 1.04) | < 0.001 | _____________ | | - |
| R1 (vs R0) | 1.72 (1.04 – 2.84) | 0.033 | _____________ | | - |
| Positive transection margin | 1.34 (0.71 – 2.55) | 0.37 |  | |  |
| Positive posterior margin | 1.1 (0.71 – 1.69) | 0.67 |  | |  |
| Positive anterior surface | 2.49 (1.57 – 3.94) | < 0.001 | 3.14 (1.61 – 6.16) | | 0.001 |
| > 1 positive margins/surfaces | 1.8 (1.1 – 2.96) | 0.02 | _____________ | | - |
| Splenic vein invasion | 1.49 (0.81 – 2.72) | 0.19 |  | |  |
| Vascular invasion | 1.98 (1.2 – 3.26) | 0.007 | _____________ | | - |
| Perineural invasion | 2.86 (1.24 – 6.59) | 0.014 | _____________ | | - |
| Adjuvant chemotherapy | 0.54 (0.35 - 0.83) | 0.006 | 0.43 (0.21 – 0.84) | | 0.014 |

c-statistic = 0.75

**Supplementary table 5. Tumor-specific parameters associated with involvement of the anterior pancreatic surfaces.**

| **Variables** | **Anterior +**  **(n=35)** | **Anterior –**  **(n=89)** | **p-value** |
| --- | --- | --- | --- |
| Tumor size (mm), mean (SD) | 48.1 (15.8) | 41.8 (19.1) | 0.08 |
| pT stage, n (%) |  |  | 0.24 |
| pT1 | 1 (2.9%) | 11 (12.4%) |  |
| pT2 | 12 (34.3%) | 33 (37.1%) |  |
| pT3 | 22 (62.9%) | 45 (50.6%) |  |
| pN stage, n (%) |  |  | 0.009 |
| pN0 | 4 (11.4%) | 33 (37.1%) |  |
| pN1 | 16 (45.7%) | 36 (40.4%) |  |
| pN2 | 15 (42.9%) | 20 (22.5%) |  |
| Poor differentiation, n (%) † | 16 (48.5%) | 22 (26.8%) | 0.026 |
| Vascular invasion, n (%) | 30 (85.7%) | 58 (65.2%) | 0.023 |
| Lymphatic invasion, n (%) | 26 (74.3%) | 58 (65.2%) | 0.33 |
| Perineural invasion, n (%) | 33 (94.3%) | 77 (86.5%) | 0.35 |
| Positive posterior margin, n (%) | 20 (57.1%) | 49 (55.1%) | 0.83 |
| > 1 positive margin/surface, n (%) | 20 (57.1%) | 14 (15.7%) | < 0.001 |

† not applicable for tumors treated with preoperative chemotherapy
